# High-Dose Vitamin D Supplementation in Pregnancy and Neurodevelopment in Childhood: A Prespecified Secondary Analysis of a Randomized Clinical Trial

**DOI:** 10.1001/jamanetworkopen.2020.26018

**Published:** 2020-12-08

**Authors:** Laerke Sass, Rebecca Kofod Vinding, Jakob Stokholm, Elín Bjarnadóttir, Sarah Noergaard, Jonathan Thorsen, Rikke Bjersand Sunde, John McGrath, Klaus Bønnelykke, Bo Chawes, Hans Bisgaard

**Affiliations:** 1 Copenhagen Prospective Studies on Asthma in Childhood, Herlev and Gentofte Hospital, University of Copenhagen, Copenhagen, Denmark; 2 Department of Pediatrics, Slagelse Hospital, Slagelse, Denmark; 3 Queensland Centre for Mental Health Research, The Park Centre for Mental Health, Wacol, Australia; 4 Queensland Brain Institute, University of Queensland, St Lucia, Australia; 5 National Centre for Register-Based Research, Department of Economics and Business Economics, Aarhus University, Aarhus, Denmark

## Abstract

**Importance:**

Observational studies have reported an association between high maternal vitamin D levels and improved neurodevelopment in offspring, but no randomized clinical trial (RCT) has investigated these observations.

**Objective:**

To determine whether high-dose vitamin D supplementation during pregnancy improves offspring neurodevelopment from birth to age 6 years.

**Design, Setting, and Participants:**

This prespecified secondary analysis of a double-blinded, placebo-controlled RCT of high-dose vitamin D_3_ supplementation vs standard dose during the third trimester of pregnancy was conducted in the unselected prospective mother-child birth cohort at a single-center research unit in Denmark as part of the Copenhagen Prospective Studies on Asthma in Childhood 2010 (COPSAC-2010). Participants included pregnant women; women with vitamin D intake greater than 600 IU/d or an endocrine, heart, or kidney disorder, and those who did not speak Danish fluently were excluded. Neurodevelopmental assessments for offspring of these women were performed at ages 0 to 6 years. Children born prematurely (gestational week <37), with low birth weight (<2500 g), or with a neurological disease affecting neurodevelopment were excluded. Data were analyzed from August 2019 to February 2020.

**Interventions:**

High-dose (ie, 2800 IU/d) vs standard dose (ie, 400 IU/d) vitamin D_3_ supplementation from pregnancy week 24 until 1 week after birth.

**Main Outcomes and Measures:**

The primary outcome of interest was cognitive development assessed at 2.5 years using the Bayley Scales of Infant and Toddler Development. Other neurodevelopmental outcomes included age of motor milestone achievement (Denver Developmental Index and World Health Organization milestone registration), language development (MacArthur-Bates Communicative Development Inventories), general neurodevelopment at age 3 years (Ages and Stages Questionnaire), and emotional and behavioral problems at age 6 years (Strengths and Difficulties Questionnaire).

**Results:**

Among 623 women randomized, 315 were randomized to high-dose vitamin D_3_ and 308 were randomized to standard dose placebo. A total of 551 children were evaluated from birth to age 6 years, (282 [51.2%] boys; 528 [95.8%] White), with 277 children in the high-dose vitamin D_3_ group and 274 children in the standard dose group. There was no effect of the high-dose compared with standard dose of vitamin D_3_ supplementation during pregnancy on offspring achievement of motor milestones (β = 0.08 [95% CI, −0.26 to 0.43]; *P* = .64), cognitive development (score difference: 0.34 [95% CI, −1.32 to 1.99]; *P* = .70), general neurodevelopment (median [IQR] communication score: 50 [50-55] vs 50 [50-55]; *P* = .62), or emotional and behavioral problems (odds ratio, 0.76 [95% CI, 0.53 to 1.09]; *P* = .14). There was no effect on language development expressed by the word production at 1 year (median [IQR], 2 [0-6] words vs 3 [1-6] words; *P* = .16), although a decreased word production was apparent at 2 years in children in the high-dose vitamin D_3_ group (median [IQR], 232 [113-346] words vs 253 [149-382.5] words; *P* = .02).

**Conclusions and Relevance:**

In this prespecified secondary analysis of an RCT, maternal high-dose vitamin D_3_ supplementation during the third trimester of pregnancy did not improve neurodevelopmental outcomes in the offspring during the first 6 years of life. These findings contribute essential information clarifying the effects of prenatal exposure to vitamin D on neurodevelopment in childhood.

**Trial Registration:**

ClinicalTrials.gov Identifier: NCT00856947

## Key Points

**Question:**

Does maternal high-dose vitamin D supplementation in the third trimester of pregnancy improve offspring neurodevelopment in the first 6 years of life?

**Findings:**

This prespecified secondary analysis of a randomized clinical trial of vitamin D_3_ supplementation during pregnancy and offspring neurodevelopment among 551 children showed no effect on neurodevelopment in the first 6 years of life, except for an isolated negative effect on language development at age 2 years in the high-dose compared with standard dose group.

**Meaning:**

This secondary analysis of a randomized clinical trial found that maternal high-dose vitamin D_3_ supplementation during pregnancy did not improve neurodevelopmental outcomes in the offspring during the first 6 years of life compared with the standard recommended dose of vitamin D.

## Teaser

This prespecified secondary analysis of a randomized clinical trial examines neurodevelopmentdal outcomes among offspring of women using high-dose vs standard dose vitamin D_3_ supplementation during pregnancy.

## Introduction

Vitamin D deficiency is a major global health problem affecting people at all ages and in all racial/ethnic groups.^1,2,3,4,5^ Vitamin D deficiency is prevalent among pregnant women,^2,6,7,8,9^ and since fetal and newborn vitamin D status is almost completely dependent on vitamin D from the mother, an adequate maternal level is pivotal.^10,11,12,13^ Vitamin D is an essential micronutrient and a neuroactive steroid that plays an important role in the development of the brain.^14,15,16^ Animal models of developmental vitamin D deficiency have found altered brain structure and behavior, which are thought to be mediated by various mechanisms affecting neurotransmission, neuronal differentiation, gene transcription, and immunological modulation.^16,17,18^

Several observational studies have found associations of prenatal vitamin D deficiency with a series of neurodevelopmental and psychiatric diseases, such as attention deficit/hyperactivity disorder, autism spectrum disorder, and schizophrenia.^19,20,21,22,23,24,25^ Hence, adequate prenatal vitamin D levels may be important not only to assure proper brain development but also for the maintenance of mental functions later in life.^18,26^

Results from observational studies on maternal levels of vitamin D during pregnancy and various neurodevelopmental outcomes are inconsistent.^27,28,29,30,31,32,33,34,35,36,37,38^ However, recent meta-analyses by Tous et al^39^ and García-Serna and Morales^40^ of observational studies found a positive association between higher maternal vitamin D levels and improved cognitive development in the offspring. Furthermore, to our knowledge, no previous randomized clinical trial (RCT) has investigated the effect of vitamin D supplementation during pregnancy on offspring neurodevelopment.^39,40,41^

Here, we present a secondary analysis of an RCT investigating the effect of high-dose vs standard dose vitamin D_3_ supplementation during the third trimester of pregnancy among women enrolled in the population-based Copenhagen Prospective Studies on Asthma in Childhood 2010 (COPSAC-2010) mother-child birth cohort.

## Methods

### Study Design

This study is a prespecified secondary analysis of a double-blinded, placebo-controlled RCT of high-dose vs standard dose vitamin D_3_ supplementation given to pregnant Danish women enrolled in the COPSAC-2010 cohort, which consists of 738 pregnant women and their 700 children followed prospectively with deep clinical phenotyping through childhood. Owing to a delay in ethical approval, 623 of 738 recruited women participated in the vitamin D_3_ trial. The women were recruited between March 4, 2009, and November 17, 2010, and their children were followed prospectively by research pediatricians with in-depth neurodevelopmental assessment at the COPSAC single-center research unit. The pregnant women were seen twice during pregnancy: at their first visit at week 24, and at week 36. The children attended the COPSAC research unit for 12 prescheduled visits until age 6 years; at 1 week, 1 month, 3 months, 6 months, then half-yearly until age 3 years, and yearly thereafter until age 6 years.

The COPSAC-2010 study was approved by the Ethics Committee of Copenhagen with a separate approval for the high-dose Vitamin D_3_ RCT during pregnancy from the Danish Health and Medicines Authority and the Danish Data Protection Agency. Parents gave oral and written informed consent before enrollment. The study is reported according to the Consolidated Standards of Reporting Trials (CONSORT) reporting guideline.

### Study Participants

Pregnant women living in Zealand, Denmark, were recruited after their initial pregnancy visit at the general physician^42,43^ and were invited to the COPSAC clinic during week 24 of pregnancy. We excluded women who did not speak Danish fluently or had a vitamin D intake greater than 600 IU/d or any endocrine, heart, or kidney disorder.^42^ The children were included in the COPSAC-2010 cohort at age 1 week.

Neurodevelopment was assessed during the first 6 years of life, excluding children born prematurely (gestational week <37), with a low birth weight (<2500g), or with a neurological disease affecting neurodevelopment.^44,45,46^ Skin color is determinative for vitamin D obtained from sun exposure, so information on race was acquired by asking the parent. Race was defined as White or non-White.

### Study Intervention

The pregnant women were randomized 1:1 to receive either 2400 IU of vitamin D_3_ per day or matching placebo. Additionally, all women were instructed to continue the 400 IU vitamin D_3_ supplementation daily recommended throughout pregnancy by the Danish National Board of Health. Hence, this study compared high-dose (2800 IU/d) vs standard dose (400 IU/d) of maternal vitamin D_3_ supplementation. The women were instructed to take the tablets from randomization at the first visit to the COPSAC research unit at week 24 and until 1 week after birth. The study was double-blinded until the youngest child turned 3 years, and investigator-blinded at all subsequent visits. The pregnant women simultaneously participated in a double-blinded RCT of 2.4 g n-3 long-chain polyunsaturated fatty acids (n-3 LCPUFA) supplementation daily during the third trimester of pregnancy.^42,47^

### Outcome Assessments

The primary outcome of the vitamin D_3_ RCT was asthma or persistent wheeze at ages 0 to 3 years,^43,48^ while neurodevelopment was a predetermined secondary outcome. During the first years of life, achievement of motor milestones was monitored prospectively by the parents using a registration form based on Denver Developmental Index^49^ and World Health Organization milestone registration.^50^ Language development at ages 1 and 2 years was assessed by MacArthur-Bates Communicative Development Inventories (MB-CDI) completed by the parents.^51^ Cognitive development was evaluated with the cognitive composite score obtained from the Bayley Scales of Infant and Toddler Development, Third edition (Bayley-III)^52^ conducted at age 2.5 years.^46^ The test was performed by trained clinicians, video recorded, and reevaluated by a person not participating in the interviews. At age 3 years, general neurodevelopment was assessed by the Ages and Stages Questionnaire (ASQ-3)^53^ completed by the parents. Behavioral and emotional problems at age 6 years were determined using the parent version of the extended Strengths and Difficulties Questionnaire (SDQ) for children aged 4 to 10 years.^54,55,56^ Additional information of the neurodevelopmental instruments used, including information of validation, scoring, and interpretation, are available in the eAppendix in Supplement 2.

### Statistical Analysis

Differences between the high-dose vitamin D_3_ supplementation and standard dose supplementation groups were estimated by *t* test for continuous variables and χ^2^ test for categorical variables. We used probabilistic principal component (PC) analysis to generate PCs for the milestone data because of a high degree of collinearity. We presumed that missing values were missing at random. The PCs were used to assess whether an overall effect of the vitamin D_3_ intervention on milestone development was present, followed by subsequent analyses of the individual milestones, all analyses were elaborated using linear regression models.

The effect of the vitamin D_3_ intervention on language development was assessed by quasi Poisson regression models. The intervention’s effect on the Bayley-III cognitive composite score was analyzed using linear regression models, while the effect on ASQ-3 general neurodevelopment was assessed by Wilcoxon rank sum test.

The primary statistical approach for the SDQ was scores for total difficulties, divided into 2 groups: children with fewer or milder problems (values ≤ median) and children with more severe problems (values > median). The scores rating the functional impact of existing emotional and behavioral problems were grouped according to no or any impact. The effect of the vitamin D_3_ intervention on the SDQ scores was determined using logistic regression models. Additionally, the effect of the vitamin D_3_ intervention on the total difficulties score as a continuous variable was estimated using linear regression models.

Interaction analyses between the vitamin D_3_ and n-3 LCPUFA interventions on all neurodevelopmental outcomes were conducted using linear regression models adding the cross product of vitamin D_3_ × n-3 LCPUFA allocation. Likewise, a possible sex interaction was investigated with linear regression models. However, before unblinding of the RCT at 3 years, it was decided to stratify all analyses for sex regardless sex interaction. This decision was based on known differences in brain development between girls and boys.^57,58^

All neurodevelopmental outcomes were analyzed unadjusted and adjusted using the same statistical analyses models and reporting the multivariate adjusted results. We adjusted for maternal preintervention serum vitamin D_3_ levels, n-3 LCPUFA RCT allocation, season of birth, and in the overall analyses additionally for sex.

No adjustment for multiple testing was performed. Missing data were treated as missing observations, except in the milestone probabilistic PC analysis. Data processing was conducted using R statistical software version 3.5.2 (R Project for Statistical Computing).

Additional methodological details are outlined in previous studies based on the same cohort^42,43,46^ and in the Trial Protocol in Supplement 1. *P* values were 2-sided, and statistical significance was set at .05. Data were analyzed from August 2019 to February 2020.

## Results

### Baseline Characteristics

Among 623 women who were 24 weeks pregnant and living in Zealand, Denmark, 315 were randomized to receive high-dose vitamin D_3_ supplementation and 308 were randomized to receive the standard-dose vitamin D_3_ placebo. Of these, 40 women (6.4%) withdrew from the study before their child was born, resulting in 587 children, including 4 twin pairs. A total of 36 children (6.1%) were excluded from the study, including 8 children without any assessment of neurodevelopmental outcomes, 4 children with a neurological disease, 19 children who were born preterm, and 5 children with low birth weight, leaving 551 children eligible for analyses, with 277 children in the high-dose vitamin D_3_ group and 274 children in the standard dose group (Figure). Of these children, 282 were boys (51.2%), and 528 (95.8%) were White. The children had a mean (SD) birthweight of 3.60 (0.48) kg, a median (interquartile range [IQR]) gestational age of 40.1 (39.3 to 41.1) weeks, and 243 (44.1%) were first-born.

**Figure.**
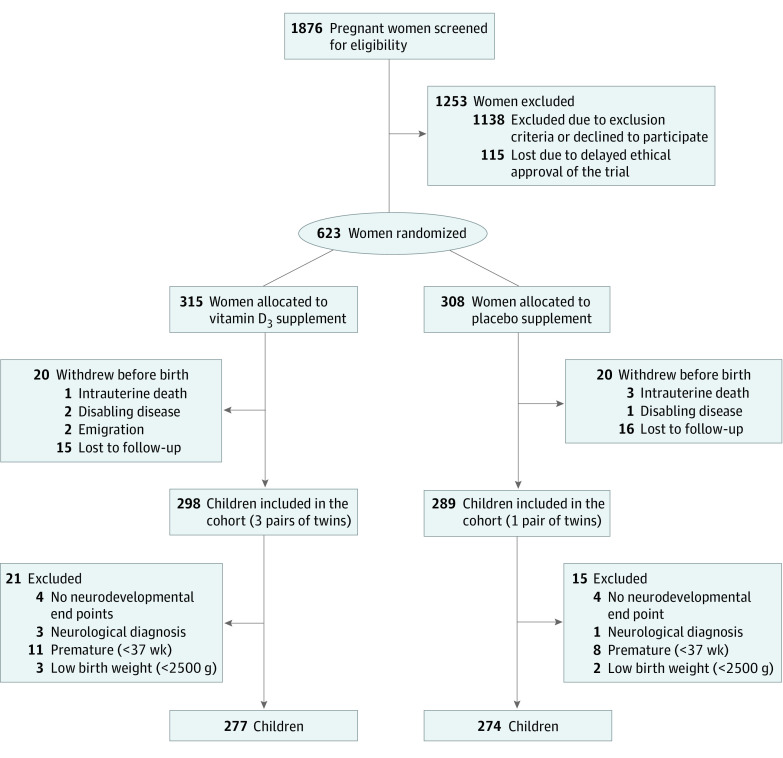
CONSORT Flowchart of Study Participants

The baseline characteristics of the pregnant women and their children are shown in eTable 1 in Supplement 2. There were no differences between the high-dose and standard dose vitamin D_3_ groups in any of the characteristics. The intervention resulted in increased maternal serum vitamin D levels 1 week after birth (mean [SD], 43.18 [14.13] ng/mL vs 28.94 [12.47] ng/mL; mean difference, 14.24 [95% CI, 12.00 to 16.48] ng/mL; *P* < .001).

Adherence to the vitamin D_3_ intervention was estimated by counting the capsules returned by the mothers at the end of the intervention period. The adherence, defined as an intake of more than 80% of the capsules provided, was 74% with no significant difference between the two groups.^43^ The RCT safety profile has previously been reported.^43^

### Achievement of Motor Milestones

A total of 520 children (94.4%) had a recorded age at milestone achievements. The probabilistic PC analysis containing all the 13 motor milestone in 1 model showed that PC1, mainly consisting of late gross motor milestones (ie, crawling, walking, and standing), explained 37% of the total data variation, while PC2, primarily constituting early milestones (ie, smiling, head lifting, and sitting with support), explained 16% of the total variation in the milestone achievements (eFigure in Supplement 2). There was no significant difference in the age of achievement of motor milestones between children in the high-dose and standard dose vitamin D_3_ supplementation groups for PC1 (β = 0.08 [95% CI, −0.26 to 0.43]; *P* = .64) or PC2 (β = 0.12 [95% CI, −0.10 to 0.35]; *P* = .28) (Table 1). Likewise, none of the 13 individual milestones were affected by high-dose vitamin D_3_ supplementation in the overall analyses nor when stratified for sex (eTable 2 in Supplement 2).

**Table 1.** Effect of Vitamin D_3_ Supplementation During Pregnancy on PC Analyses of Motor Milestones

Outcome^a^	Vitamin D_3_ dose, mean (SD)		Unadjusted (n = 520)		Adjusted (N = 517)^b^	
	High (n = 261)	Standard (n = 259)	Effect, β (95% CI)	*P* value	Effect, β (95% CI)	*P* value
PC1^c^	−0.04 (2.0)	−0.11 (2.0)	0.07 (−0.27 to 0.42)	.67	0.08 (−0.26 to 0.43)	.64
Girls	0.19 (2.2)	−0.13 (1.9)	0.32 (−0.18 to 0.82)	.21	0.30 (−0.20 to 0.81)	.24
Boys	−0.24 (1.8)	−0.09 (2.1)	−0.15 (−0.62 to 0.32)	.53	−0.13 (−0.61 to 0.34)	.58
PC2^d^	−0.02 (1.4)	−0.13 (1.2)	0.11 (−0.11 to 0.34)	.34	0.12 (−0.10 to 0.35)	.28
Girls	−0.04 (1.4)	−0.14 (1.3)	0.10 (−0.24 to 0.43)	.56	0.12 (−0.22 to 0.45)	.49
Boys	−0.01 (1.3)	−0.12 (1.2)	0.12 (−0.18 to 0.42)	.44	0.16 (−0.14 to 0.47)	.30

Abbreviation: PC, principal component.

^a^ Lower PC scores indicate younger age at milestone achievement.

^b^ Adjusted for maternal preintervention serum vitamin D_3_ levels, n-3 LCPUFA RCT allocation, season of birth, and for overall analyses additionally for sex. Three mothers were missing preintervention serum vitamin D_3_ levels.

^c^ Includes late gross motor milestones (ie, crawling, walking, and standing).

^d^ Includes early milestones (ie, smiling, head lifting, and sitting with support).

### Language Development

Children with a language other than to Danish spoken at home were excluded from our language analyses, hence 284 children (51.5%) had their language development evaluated at age 1 year. The high-dose vitamin D_3_ supplementation during pregnancy did not alter the vocabulary at age 1 year compared with that of the control group estimated by the word production score of the MB-CDI (median [IQR], 2 [0 to 6] words vs 3 [1 to 6] words; *P* = .16) (Table 2). At age 2 years, the word production of 393 children (71.3%) was assessed. The children in the high-dose vitamin D_3_ supplementation group, compared with the control group, had a reduction in word production compared with the children in the standard dose group (median [IQR]; 232 [113.0 to 346.0] vs 253 [149.0 to 382.5]; *P* = .02), showing similar direction in girls and boys (Table 2). The lower number of children in the 1-year language test compared with the 2-year test was caused by late implementation of the MB-CDI test. This delay resulted in a proportion of children being too old for the test at implementation time (eTable 3 in Supplement 2).

**Table 2.** Effect of Vitamin D_3_ Supplementation During Pregnancy on Language Scores

	Median (IQR)		Risk ratio *P* value	
	High dose	Standard dose	Unadjusted	Adjusted^a^
**Age 1 y language test**				
Children, No.	137	147	284	281
Word production	2 (0-6)	3 (1-6)	.13	.16
Girls	3 (1-7)	3 (1-6)	.63	.58
Boys	1 (0-5)	4 (1-7)	.11	.13
**Age 2 y language test**				
Children, No.	199	194	393	390
Word production	232.0 (113.0-346.0)	253.0 (149.0-382.5)	.01	.02
Girls	274.5 (156.8-383.8)	307.0 (187.2-412.2)	.12	.14
Boys	190.0 (66.0-320.0)	195.5 (94.8-344.3)	.09	.14

Abbreviation: IQR, interquartile range.

^a^ Adjusted for maternal preintervention serum vitamin D_3_ levels, n-3 LPUFA RCT allocation, season of birth, and for overall analyses additionally for sex. Three mothers were missing preintervention serum vitamin D_3_ results.

### Cognitive Development

Cognitive development was a priori defined as the primary neurodevelopmental outcome of the RCT (eTable 4 in Supplement 2). A total of 503 children (91.3%) completed the cognitive composite score of the Bayley-III at age 2.5 years. There was no difference in the cognitive composite score between children in the high-dose compared with standard dose vitamin D_3_ groups, neither overall (score difference: 0.34 [95% CI, −1.32 to 1.99]; *P* = .70) nor when stratifying for sex (Table 3).

**Table 3.** Effect of Vitamin D_3_ Supplementation During Pregnancy on the Cognitive Composite Score of the Bayley-III Test

Outcome	Vitamin D_3_ dose, mean (SD)		Unadjusted (n = 503)		Adjusted (n = 501)^a^	
	High (n = 245)	Standard (n = 258)	Effect, β (95% CI)	*P* value	Effect, β (95% CI)	*P* value
Cognitive score	104.7 (9.5)	104.5 (9.5)	0.24 (−1.42 to 1.89)	.78	0.34 (−1.32 to 1.99)	.70
Girls	105.6 (9.6)	105.4 (10.3)	0.15 (−2.35 to 2.64)	.91	0.15 (−2.39 to 2.69)	.91
Boys	104.0 (9.3)	103.5 (8.4)	0.46 (−1.71 to 2.63)	.68	0.79 (−1.40 to 2.98)	.48

^a^ Adjusted for maternal preintervention serum vitamin D_3_ levels, n-3 long-chain polyunsaturated fatty acid RCT allocation, season of birth, and for overall analyses additionally for sex. Two mothers were missing preintervention serum vitamin D_3_ levels.

### General Neurodevelopment

At age 3 years, 405 children (73.5%) had a completed ASQ-3 test. There was no effect of the high-dose vitamin D_3_ supplementation on the general neurodevelopment reflected in the 5 scores of the ASQ-3. In sex-stratified analyses, girls in the high-dose vitamin D_3_ supplementation group had decreased gross motor skills compared with the standard dose group (median [IQR], 57.5 [55 to 60] vs 60.0 [55 to 60]; *P* = .04), whereas there were no differences among boys (eTable 5 in Supplement 2).

### Emotional and Behavioral Problems

At age 6 years, parents of 496 children (90.0%) completed the SDQ. The total difficulties score from the SDQ was not affected by the vitamin D_3_ supplementation, neither when analyzed continuously (score difference: −0.02 [95% CI, −0.86 to 0.82]; *P* = .96) nor as a binary outcome (OR, 0.76 [95% CI, 0.53 to 1.09]; *P* = .14). Additionally, there was no effect on any of the subscales of the total difficulties score when analyzed separately. Furthermore, there was no effect of the supplementation on the impact score (OR, 1.33 [95% CI, 0.76 to 2.30]; *P* = .30) (Table 4).

**Table 4.** Effect of Vitamin D_3_ Supplementation During Pregnancy on Outcomes of the Strengths and Difficulties Questionnaire

Outcome	Vitamin D_3_ dose, No. (%)		Unadjusted (n = 496)		Adjusted (n = 493)^a^	
	High (n = 246)	Standard (n = 250)	OR (95% CI)	*P* value	OR (95% CI)	*P* value
**Total difficulties score (above vs below median)**						
All children	109 (44.3)	126 (50.4)	0.78 (0.55-1.11)	.18	0.76 (0.53-1.09)	.14
Girls	48 (19.5)	61 (24.4)	0.78 (0.47-1.29)	.33	0.73 (0.43-1.23)	.24
Boys	61 (24.8)	65 (26.0)	0.78 (0.47-1.27)	.32	0.77 (0.47-1.27)	.30
**Impact score (any vs none)**						
All children	36 (14.6)	28 (11.2)	1.36 (0.80-2.32)	.26	1.33 (0.76-2.30)	.30
Girls	16 (6.5)	9 (3.6)	2.12 (0.91-5.20)	.09	1.97 (0.83-4.95)	.13
Boys	20 (8.1)	19 (7.6)	0.99 (0.50-1.96)	.97	0.97 (0.48-1.99)	.93

Abbreviation: OR, odds ratio.

^a^ Adjusted for maternal preintervention serum vitamin D3 levels, n-3 long-chain polyunsaturated fatty acid RCT allocation, season of birth, and for overall analyses additionally for sex. Three mothers are missing preintervention serum vitamin D_3_ levels.

There were no significant interactions between the vitamin D_3_ and n-3 LCPUFA interventions on any of the neurodevelopmental outcomes. Likewise, there was no significant sex interaction on any of the neurodevelopmental outcomes.

Analyzing data without excluding any children yielded comparable results (eTable 6 in Supplement 2). Furthermore, to reassure an elimination of any possible effect of the n-3 LCPUFA supplementation, we analyzed the data of 281 women who did not simultaneously receive n-3 LCPUFA; this analysis did not change our findings, except that there was no longer a negative effect of the high-dose vitamin D_3_ on word production at age 2 years (median [IQR], 227.0 [79.0 to 347.5] words vs 239.0 [116.2 to 411.2] words; *P* = .17) (eTable 7 in Supplement 2).

## Discussion

This prespecified secondary analysis of a large-scale RCT of high-dose vitamin D_3_ supplementation during the third trimester of pregnancy showed no consistent effects on a multitude of neurodevelopmental outcomes during the first 6 years of life compared with standard dose, and particularly, no beneficial effects were found.

To our knowledge, this study is the first double-blinded RCT of vitamin D_3_ supplementation during pregnancy and offspring neurodevelopment.^39,40,41^ Therefore, this study contributes with essential information clarifying the effects of prenatal exposure to vitamin D on neurodevelopment in childhood.

Among the most important strengths of this study are the size of the study population, the recruitment strategy enabling enrollment from the general population, the longitudinal design allowing neurodevelopmental assessments at numerous time points during the children’s first 6 years of life, and the high adherence and follow-up rates.

Neurodevelopment was assessed using several validated tools^49,50,51,52,53,54,55,56^ investigating different aspects, including achievement of motor milestones, language development, cognitive development, and emotional and behavioral problems. These standardized tests are used worldwide,^59,60,61^ which makes them very suitable for comparison among studies across countries and enhances the generalizability of our results. The cognitive composite score of the Bayley-III is an early marker of long-term cognitive functioning and is highly correlated with the full-scale IQ^62^ and global conceptual ability score later in childhood.^63^ The Bayley-III was performed by trained clinicians assuring consistency in the testing procedures. The test was video recorded and reevaluated by a person not participating in the interviews, improving the interrater reliability of the score for each child. The 8 scheduled visits to our clinical research unit until age 2.5 years assured familiarity and confidence between children and clinicians, improving cooperation during tests and thereby the quality of the obtained data.

### Interpretation

In line with our null result for cognitive development, several observational studies report no association of maternal vitamin D levels during pregnancy,^27,28,29,30,31,32^ cord blood vitamin D levels,^33^ or both^34^ with cognition. One observational study described a positive association of maternal vitamin D levels in pregnancy with motor development and cognition,^35^ while another study found that IQ at age 7 years was positively associated with either maternal vitamin D levels during pregnancy or cord blood vitamin D levels, although the effect estimates were very small.^36^ However, a 2020 meta-analysis^39^ found that children born to vitamin D insufficient mothers had lower cognitive scores, while another meta-analysis^40^ found an association between increasing vitamin D concentrations in maternal blood during pregnancy or cord blood and improved cognitive development in offspring. Our results showed no effect of the high-dose vitamin D_3_ supplementation on motor milestone achievement or behavioral and emotional problems, which is supported by observations from several prospective studies.^28,31,34,36,37^

Unexpectedly, we observed that the children of the high-dose vitamin D_3_ supplementation group had an impairment in word production at age 2 years and that the girls had a reduction in gross motor skills at age 3 years. These isolated findings in otherwise cognitively intact children should be interpreted cautiously. The findings were not in the predicted direction and are not consistent with related observational studies^29,33,38^ and a 2020 meta-analysis,^39^ which reported an association of higher maternal or cord blood vitamin D concentrations with improved language skills. Some observational studies have not found any association between maternal vitamin D levels in pregnancy and language development,^28,31^ but to our knowledge, no studies have reported negative associations between prenatal vitamin D exposure and language skills.^39,40^ Still, it is feasible that high-dose vitamin D_3_ supplementation during pregnancy is associated with adverse brain outcomes. A Danish case-control study^19^ based on neonatal dried blood spots reported a U-shaped association between neonatal vitamin D status and risk of schizophrenia; both low and high vitamin D concentrations were associated with an increased risk of schizophrenia compared with the reference category. However, the underlying mechanism of a potential adverse effect of high vitamin D exposure on language and other neurodevelopmental outcomes is unknown.

It is also possible that our result of a negative effect of the high-dose vitamin D_3_ pregnancy supplementation on word production at age 2 years is a spurious finding caused by a type 1 error (ie, rejection of a true null hypothesis). Applying a correction for multiple comparisons would alleviate the risk of type 1 errors but might increase the risk of type 2 errors (ie, nonrejection of a false null hypothesis). Hence, since this study is a prespecified secondary analysis of an RCT, we chose not to adjust for multiple testing. However, if we do adjust for multiple testing with Bonferroni, the statistically significant effect of high-dose vitamin D_3_ on word production at age 2 years is no longer present.

Furthermore, in a subanalysis in the group of women who did not simultaneously receive n-3 LCPUFA, we can, despite a markedly reduced number, study the isolated effect of the high-dose vitamin D_3_ supplementation. In this analysis, we did not find a significant effect on word production at age 2 years.

The ongoing longitudinal follow-up of the COPSAC-2010 cohort with a range of cognitive and neuropsychiatric assessments at 10 years will enable us to further examine the effects of the high-dose vitamin D_3_ supplementation.

Through the past decade, several observational studies^29,33,35,36,38^ have reported an association between prenatal vitamin D levels and neurodevelopment in the children, generating the hypothesis that vitamin D_3_ supplementation during pregnancy would enhance offspring neurodevelopment. However, since vitamin D status is strongly influenced by lifestyle factors, it is almost impossible to account for confounding from these in observational studies. To our knowledge, our study is the first RCT of vitamin D_3_ supplementation during pregnancy and offspring neurodevelopment, and we found no evidence to support this hypothesis.

Besides the fact that our study is an RCT, some of the discrepancies between our results and the findings of previous studies might be caused by a diversity in assessment methods, different age at assessment, lower follow-up rates and variation in choice of confounders for adjustment in the observational studies or differences between vitamin D levels in the mothers.

Our findings suggest that the standard dose (ie, 400 IU/d) of maternal vitamin D_3_ supplementation during pregnancy is adequate for a healthy neurodevelopment among children in the Danish population.

The lack of positive effects from the high-dose vitamin D_3_ supplementation on neurodevelopment might be explained by the timing of the intervention: although the most pronounced period of brain growth and development is during the third trimester of pregnancy, the formation of essential brain structures starts during early pregnancy.^11,38^ However, from this RCT, we are unable to establish whether an earlier start of the intervention or another dose would have provided positive results. Nonetheless, it is possible that initiation of the vitamin D_3_ supplementation earlier in pregnancy might affect the offspring’s neurodevelopment, and future RCTs should take the timing of supplementation into consideration. Furthermore, future studies could possibly benefit from a higher vitamin D_3_ supplementation dose, as doses up to 5000 IU/d are considered safe according to a 2018 meta-analysis of 24 RCTs.^64^

Finally, it can be speculated that vitamin D_3_ supplementation during pregnancy might only have advantageous effects on offspring neurodevelopment in populations with lower levels of vitamin D, such as individuals with lower fish-intake,^65^ higher levels of skin pigmentation, limited sunlight exposure owing to extensive skin coverage, or increased use of skin protection.^5,40^

### Limitations

This study has some limitations, including missing data from early language development and ASQ-3 owing to late implementation of these tests. Another possible limitation is potential recall bias owing to retrospective registration of motor milestones for some of the children. Nevertheless, studies have shown an excellent correlation between motor milestones assessed by a pediatric neurologist and parental remembrance of the age of milestone achievement 2 years later.^66^ We have no IQ assessments of the parents, but comprehensive parental data, including aspects associated with IQ, were available (eg, educational level and income), which showed no difference between the intervention groups. Additionally, our study population consisted almost entirely of mother-child pairs of European ancestry, and it is therefore not possible to extrapolate our findings to other racial/ethnic populations.

It is also important to note that at enrollment, very few mothers included in this study had vitamin D serum concentrations less than 12 ng/mL, which is a commonly used definition of vitamin D deficiency. These low numbers precluded stratified analysis in this subgroup, hence our study could not address if prenatal vitamin D_3_ supplementation is specifically beneficial to offspring of mothers with vitamin D deficiency.

Regarding analytic approach, we chose to analyze the SDQ scores as split by median, which could be considered a limitation of the study because of the reduction in the obtained information. However, we also analyzed the data continuously, which similarly showed no difference between the intervention groups. Furthermore, in a cohort setting of children with an expected normal neurodevelopment, it is a valuable way of investigating any gross differences between groups.

## Conclusions

In this prespecified secondary analysis of an RCT, maternal high-dose vitamin D_3_ supplementation during the third trimester of pregnancy did not improve neurodevelopment in the offspring during the first 6 years of life compared with the standard recommended dose of vitamin D_3_.

## Notes

Supplement 1. **Trial Protocol**

Supplement 2. **eAppendix.** Supplemental Methods **eReferences** **eTable 1.** Characteristics of the COPSAC Mother-Child Cohort **eTable 2.** Effect of High-Dose Vitamin D_3_ Supplementation During Pregnancy on Individual Motor Milestones **eTable 3.** Overview of Children at Each Neurodevelopmental Assessment **eTable 4.** Descriptive Classification of the Cognitive Composite Score of the Bayley-III **eTable 5.** Effect of High-Dose Vitamin D_3_ Supplementation During Pregnancy on Scores From the Ages and Stages Questionnaire-3 **eTable 6.** Effect of the High-Dose Vitamin D_3_ Supplementation During Pregnancy on the Cognitive Composite Score of the Bayley-III Test Including Children Born Before 37 Gestational Weeks, Children With Low Birth Weight, and Children With a Neurological Diagnosis **eTable 7.** Language Development in Women Not Participating in the n-3 LCPUFA Trial **eFigure.** Biplot From Principal Component Analysis of All 13 Milestones

Supplement 3. **Data Sharing Statement**
