# Supplementary material for: High-Dose Vitamin D Supplementation in Pregnancy and Neurodevelopment in Childhood: A Prespecified Secondary Analysis of a Randomized Clinical Trial
Source: JAMA Netw Open. 2020 Dec 8;3(12):e2026018. doi: 10.1001/jamanetworkopen.2020.26018 (PMC7724557; doi:10.1001/jamanetworkopen.2020.26018)
Supplement: Supplement 2. — eAppendix. Supplemental Methods eReferences eTable 1. Characteristics of the COPSAC Mother-Child Cohort eTable 2. Effect of High-Dose Vitamin D3 Supplementation During Pregnancy on Individual Motor Milestones eTable 3. Overview of Children at Each Neurodevelopmental Assessment eTable 4. Descriptive Classification of the Cognitive Composite Score of the Bayley-III eTable 5. Effect of High-Dose Vitamin D3 Supplementation During Pregnancy on Scores From the Ages and Stages Questionnaire-3 eTable 6. Effect of the High-Dose Vitamin D3 Supplementation During Pregnancy on the Cognitive Composite Score of the Bayley-III Test Including Children Born Before 37 Gestational Weeks, Children With Low Birth Weight, and Children With a Neurological Diagnosis eTable 7. Language Development in Women Not Participating in the n-3 LCPUFA Trial eFigure. Biplot From Principal Component Analysis of All 13 Milestones [file jamanetwopen-e2026018-s002.pdf]

Supplemental Online Content

Sass L, Vinding RK, Stokholm J, et al. High-dose vitamin D supplementation in pregnancy and neurodevelopment in childhood: a prespecified secondary analysis of a randomized clinical trial. *JAMA Netw Open*. 2020;3(12):2026018.
doi:10.1001/jamanetworkopen.2020.26018

eAppendix. Supplemental Methods
eReferences
eTable 1. Characteristics of the COPSAC Mother-Child Cohort
eTable 2. Effect of High-Dose Vitamin D_3 Supplementation During Pregnancy on Individual Motor Milestones
eTable 3. Overview of Children at Each Neurodevelopmental Assessment
eTable 4. Descriptive Classification of the Cognitive Composite Score of the Bayley-III
eTable 5. Effect of High-Dose Vitamin D_3 Supplementation During Pregnancy on Scores From the Ages and Stages Questionnaire-3
eTable 6. Effect of the High-Dose Vitamin D_3 Supplementation During Pregnancy on the Cognitive Composite Score of the Bayley-III Test Including Children Born Before 37 Gestational Weeks, Children With Low Birth Weight, and Children With a Neurological Diagnosis
eTable 7. Language Development in Women Not Participating in the n-3 LCPUFA Trial
eFigure. Biplot From Principal Component Analysis of All 13 Milestones

This supplemental material has been provided by the authors to give readers additional information about their work.

© 2020 Sass L et al. *JAMA Network Open.*

eAppendix. Supplemental Methods

Maternal serum Vitamin D levels

The venous blood samples obtained before and after the intervention were centrifuged for 10min at 4300rpm to separate serum, and thereafter frozen at -80oC until analysis. The serum samples were transported on dry ice for duplicate analyses for 25-hydroxyvitamin D2(25(OH)-Vitamin D2) and 25(OH)-Vitamin D_3 at the Dept. of Clinical Biochemistry, Aarhus University Hospital, Denmark. Serum 25-hydroxyvitamin D levels were analysed by isotope dilution liquid chromatography-tandem mass spectrometry (LC-MS/MS)(1,2). Calibrators traceable to NIST SRM 972 (Chromsystems, DE) were used.

Mean coefficients of variation (CV) for 25(OH)-Vitamin D_3 were 6.4% and 9.1% at levels of 66.5 and 21.1nmol/L and for 25(OH)-Vitamin D_2 the CV values were 8.8% and 9.4% at levels of 41.2 and 25.3nmol/L. The average of the combined 25(OH)-Vitamin D values was calculated and used in the analysis. If both 25(OH)-Vitamin D_2 and 25(OH)-Vitamin D_3 were under the detection level, the combined value was defined as equal to 10nmol/L.

Maternal serum vitamin D levels were maintained double-blinded until the youngest child in the cohort turned 3 years.

Inclusion criteria

To be included in the vitamin D_3 RCT the following criteria should be met: women pregnant in week 22-26, living in Zealand, Denmark, speaking Danish fluently, participating in the $COPSAC_{2010}$ cohort and who were willing to let their child participate in the study subsequently.

Randomization

The randomization was performed using a computer-generated list of random numbers prepared by an external investigator with no other involvement in the trial. The women's serum vitamin D_3 level was measured (3,4) at the time of randomization and one week postpartum. Adherence was estimated from number of capsules returned.

Power calculation

The trial was originally powered according to the primary outcome asthma/persistent wheeze. Thus, the power of the prespecified secondary analysis of the RCT on neurodevelopment was calculated post-hoc based on the 503 children who completed the primary outcome from the Bayley-III: the cognitive composite score. This resulted in 80% power to detect a mean score difference of 2.38, with a standard deviation of 9.5. The power/sample size calculation and testing were based on a two sample, two tailed *t*-test with an α of 0.05.

Neurodevelopmental outcomes

The milestone development was monitored prospectively by the parents using a registration form based on The Denver Developmental Index(5) and WHO milestone registration(6). The parents were instructed to register the date when the child mastered each of the 13 predefined milestones. The following 6 gross motor milestones are derived from the WHO registration; "Sit alone", "Crawling", "Stand with help", "Walk with help", "Stand alone", and "Walk alone", while the remaining 7 milestones are from the Denver Developmental Index; "Lift head", "Smile", "Laugh", "Chest up", " Roll over", "Pincer grasp", and "Sit with support".

Based on birth date and date of milestone achievement, the achievement time in days for each of the individual milestones was quantified. A lower number of days is reflecting improvement of outcome.

For some children early milestones were registered retrospectively since the implementation of milestone registration began after the first 387 children were born.

Language assessment was performed as a web-based questionnaire filled out by the parents around the child's 1-year (CDI-WG: Words and gesticulation) and 2 years (CDI-WS: Words and Sentences) birthdays. The CDI, is a well-recognized and validated tool to assess monolingual children's lexical growth(7). The 1-year test evaluates language comprehension, early word production and gestural communication and the 2-year test assesses vocabulary, grammatical skills, syntax and morphology. The outcome word production was chosen a priori as the primary outcome from both the 1-and 2-year language tests. For the 1-year test word production is assessed by counting the number of word that the child says or does a lingual imitation of from a list of 409 words which are commonly found in the vocabulary of Danish children at that age. For the 2-year test, word production was assessed by counting the number of words the child pronounces from a list of 725 common words. Hence, higher scores reflect improvement of the outcome.

The CDI was not performed in the first 160 participants as it was implemented in the cohort after they had completed their 1-year visit. Children who were considered bilingual (regularly in contact with another language than Danish at home) where excluded from the language development analyses.

The Bayley Scales of Infant and Toddler Development, Third edition (Bayley-III)(8) performed at 2½ years(9). Bayley-III was administered by trained clinical personnel with a longstanding experience conducting pediatric examinations. Video recordings of every test were reviewed by one responsible clinician to ensure the validity of the scoring. A high cognitive score reflects an improved cognition (**eTable 6)**.

At 3 years of age the parents filled out the Danish version(10) of the ASQ-III questionnaires(11), which is a brief measure of the child's current skills and development in the areas of communication, gross motor skills, fine motor skills, problem solving, personal-social skills. The questionnaire consists of 30 items (six in each category) and each item is scored depending upon whether the child performs the item consistently (10 points), sometimes (5 points), or not yet (0 points). Scores for each area where then summed. The higher the scores the better the communicative/gross motor/fine motor/problem-solving/personal-social skills does the child have.
The ASQ-III was not performed in the first 124 participants as they were too old when the testing was implemented (>3 years and 3 months).

At 6 years of age the parents completed the Danish version(12,13) of the extended Strength and Difficulties Questionnaire (SDQ)(14) for children aged 4-10. SDQ is a brief behavioral screening questionnaire evaluating children's mental well-being, function and behavior consisting of 25 items reflecting both positive and negative attributes as well as an impact supplement. The 25 items are divided into 5 scales each containing 5 items and generating scores for emotional problems, conduct problems, hyperactivity-inattention, peer problems and prosocial behavior. The first four scores comprise a total difficulties score. Each item is scored from 0 to 2 point ("Not true" = 0, "Somewhat true" = 1 and "Certainly true" = 2 point). Hence, for total difficulties score and the 4 sub-scales constituting it: emotional problems, conduct problems, hyperactivity-inattention, and peer problems the lower the scores the fewer problems, while the interpretation of the Prosocial score it revers, thus the higher the Prosocial core the better. The impact supplement is only accessible by positively responding to a question regarding presence of overall difficulties, and it encompass the chronicity, distress, social impairment and burden on the parent or on the entire family(14), giving an impact score from 0-10 point. Hence, a high impact scores implies increased implication of the problems the child has. In a clinical setting the impact supplement can thereby provide fundamental information pivotal to fulfil the diagnostic criteria in the classification systems, ICD-10 and DSM-IV(13,15). Solely SDQs completed within 6 months from the child's 6-year birthday are included in the present study.

© 2020 Sass L et al. *JAMA Network Open.*

eReferences

1. Højskov CS, Heickendorff L, Møller HJ. High-throughput liquid-liquid extraction and LCMSMS assay for determination of circulating 25(OH) vitamin D3 and D2 in the routine clinical laboratory. Clin Chim Acta [Internet]. 2010 Jan [cited 2011 Oct 24];411(1–2):114–6. Available from: http://www.ncbi.nlm.nih.gov/pubmed/19850018

2. Maunsell Z, Wright DJ, Rainbow SJ. Routine isotope-dilution liquid chromatography-tandem mass spectrometry assay for simultaneous measurement of the 25-hydroxy metabolites of vitamins D2 and D3. Clin Chem. 2005 Sep;51(9):1683–90.

3. Højskov CS, Heickendorff L, Møller HJ. High-throughput liquid-liquid extraction and LCMSMS assay for determination of circulating 25(OH) vitamin D3 and D2 in the routine clinical laboratory. Clin Chim Acta. 2010 Jan;411(1–2):114–6.

4. Maunsell Z, Wright DJ, Rainbow SJ. Routine isotope-dilution liquid chromatography-tandem mass spectrometry assay for simultaneous measurement of the 25-hydroxy metabolites of vitamins D2 and D3. Clin Chem. 2005 Sep;51(9):1683–90.

5. Frankenburg W, Dodds J. The Denver developmental assessment (Denver II). Denver: University of Colorado Medical School; 1990.

6. Wijnhoven TM, de Onis M, Onyango AW, Wang T, Bjoerneboe G-EA, Bhandari N, et al. Assessment of gross motor development in the WHO Multicentre Growth Reference Study. Food Nutr Bull. 2004 Mar;25(1 Suppl):S37-45.

7. Bleses D, Vach W, Slott M, Wehberg S, Thomsen P, Madsen TO, et al. The Danish Communicative Developmental Inventories: validity and main developmental trends. J Child Lang. 2008 Aug;35(3):651–69.

8. Bayley, N. Bayley Scales of Infant and Toddler Development (Third edition),Administration Manual. Harcourt Assessment. San Antonio, TX; 2006.

9. Bjarnadóttir E, Stokholm J, Chawes B, Thorsen J, Mora-Jensen A-RC, Deleuran M, et al. Determinants of Neurodevelopment in Early Childhood - Results from the Copenhagen Prospective Studies on Asthma in Childhood (COPSAC2010) Mother-Child Cohort. Acta Paediatr. 2019 Feb 12;

10. Engel S, Tronhjem KMH, Hellgren LI, Michaelsen KF, Lauritzen L. Docosahexaenoic acid status at 9 months is inversely associated with communicative skills in 3-year-old girls. Matern Child Nutr. 2013 Oct;9(4):499–510.

11. Squires J, Bricker DD, Twombly E. Ages & stages questionnaires: a parent-completed child monitoring system. Baltimore: Paul H. Brooks Pub. Co.; 2009.

12. Obel C, Dalsgaard S, Stax H-P, Bilenberg N. [Strengths and Difficulties Questionnaire (SDQ-Dan). A new instrument for psychopathologic screening of children aged 4-16 years]. Ugeskr Laeg. 2003 Jan 27;165(5):462–5.

13. Niclasen J, Teasdale TW, Andersen A-MN, Skovgaard AM, Elberling H, Obel C. Psychometric properties of the Danish Strength and Difficulties Questionnaire: the SDQ assessed for more than 70,000 raters in four different cohorts. PLoS ONE. 2012;7(2):e32025.

14. Goodman R. The extended version of the Strengths and Difficulties Questionnaire as a guide to child psychiatric caseness and consequent burden. J Child Psychol Psychiatry. 1999 Jul;40(5):791–9.

15. Goodman R. Psychometric properties of the strengths and difficulties questionnaire. J Am Acad Child Adolesc Psychiatry. 2001 Nov;40(11):1337–45.

© 2020 Sass L et al. *JAMA Network Open.*

eTable 1. Characteristics of the COPSAC Mother-Child Cohort

	All	High-dose vitamin D_3	Standard dose vitamin D_3
**N**	551	277	274
**Maternal characteristics**			
Maternal age, years, mean (SD)	32.25 (4.33)	32.61 (4.34)	31.89 (4.30)
Maternal asthma, N (%) [a]	143 (26.0)	70 (25.3)	73 (26.6)
Social circumstances, mean (SD) [b]	0.01 (0.98)	0.12 (1.00)	-0.10 (0.95)
Smoking in pregnancy, N (%)	40 (7.3)	16 (5.8)	24 (8.8)
Alcohol intake during pregnancy, N (%)	85 (15.4)	44 (15,9)	41 (15)
Participation in long chain n-3 LPUFA RCT, N (%)	551 (100)	277 (50.3)	271 (4 9.7)
Pre-intervention serum vitamin D level, ng/mL, mean (SD)	30.60 (10.19)	30.83 (10.21)	30.36 (10.17)
Mothers with vitamin D serum levels ≥ 30 ng/mL at gestational week 24, N (%)	284 (52)	144 (52)	140 (51)
Mothers with vitamin D serum levels ≥ 20 ng/mL and < 30 ng/mL at gestational week 24, N (%)	187 (34)	88 (32)	99 (36)
Mothers with vitamin D serum levels ≥ 12ng/mL and < 20 ng/mL at gestational week 24, N (%)	64 (12)	37 (14)	27 (10)
Mothers with vitamin D serum levels < 12 ng/mL at gestational week 24, N (%)	13 (2)	6 (2)	7 (3)
**Births**			
Gestational age, weeks, median (IQR)	40.1 (39.3-41.1)	40.1 (39.1-41.1)	40.3 (39.4-41.0)
Birth order, first born, N (%)	243 (44.1)	110 (39.7)	133 (48.5)
Season of birth			
Winter, N (%)	199 (36.1)	100 (36.1)	99 (36.1)
Spring, N (%)	100 (18.1)	51 (18.4)	49 (17.9)
Summer, N (%)	114 (20.7)	58 (20.9)	56 (20.4)
Fall, N (%)	138 (25.0)	68 (24.5)	70 (25.5)
Birth weight, kg, mean (SD)	3.60 (0.48)	3.62 (0.47)	3.57 (0.49)
Apgar score at 5 min., mean (SD)	9.95 (0.28)	9.95 (0.28)	9.95 (0.28)
Intra-partum antibiotics, N (%)	162 (29.4)	83 (30.0)	79 (28.8)
Antibiotics to the child, N (%) [c]	13 (2.4)	5 (1.8)	8 (2.9)
Cesarean section, N (%)	114 (20.7)	59 (21.3)	55 (20.1)
Elective	55 (10.0)	29 (10.5)	26 (9.5)
Emergency	59 (10.7)	30 (10.8)	29 (10.6)
**Children**			
Sex, male, N (%)	282 (51.2)	145 (52.3)	137 (50.0)
Race, non-White, N (%)	23 (4.2)	10 (3.6)	13 (4.7)
**Neurodevelopmental outcomes**			
Milestone registration, N (%)	520 (94.4)	261 (94.2)	259 (94.5)
1-year language test, N (%)	284 (51.1)	137 (49.5)	147 (53.6)
2-year language test, N (%)	393 (71.3)	199 (71.8)	194 (70.8)
Bayley-III test, N (%)	503 (91.3)	245 (88.4)	258 (95.2)
ASQ test, N (%)	405 (73.5)	197 (71.1)	208 (75.9)
SDQ test, N (%)	496 (90)	246 (88.8)	250 (91.2)

© 2020 Sass L et al. *JAMA Network Open.*

Abbreviations: N=number, SD=standard deviation, CI=confidence interval, IQR (inter-quartile range)
SI conversion factor: To convert vitamin D_3 to nanomoles per liter multiply by 2.496
[a] History of doctor diagnosed asthma
[b] PCA component consisting of household income, maternal age and maternal educational level at the age of 2. The higher the value the better (equivalent higher level of education, higher level of income and older mother)
[c] Antibiotics given to the child up to 1 week after birth

© 2020 Sass L et al. *JAMA Network Open.*

eTable 2. Effect of High-Dose Vitamin D_3 Supplementation During Pregnancy on Individual Motor Milestones[a]

	High-dose Vitamin D_3	Standard dose Vitamin D_3	Unadjusted β-coefficients		Adjusted[b] β-coefficients	
	N = 261	N = 259	N = 520		N = 517	
	Mean of days (SD)		Effect (95 % CI)	P value	Effect (95 % CI)	P value
**Lift head**	37.89 (30.3)	36.57 (32.2)	0.035 (-0.13;0.20)	0.68	0.035 (-0.13;0.20)	0.68
Girls	38.63 (31.3)	36.78 (31.8)	0.049 (-0.19;0.28)	0.68	0.044 (-0.19;0.28)	0.72
Boys	37.18 (29.4)	36.34 (32.8)	0.023 (-0.22;0.27)	0.85	0.050 (-0.19;0.29)	0.68
**Smile**	36.99 (21.0)	35.08 (17.9)	1.91 (-1.80;5.62)	0.31	1.91 (-1.80;5.63)	0.31
Girls	36.29 (21.9)	34.17 (18.5)	2.12 (-3.33;7.57)	0.44	2.03 (-3.47;7.52)	0.47
Boys	37.63 (20.2)	36.07 (17.3)	1.56 (-3.54;6.67)	0.55	2.47 (-2.67;7.61)	0.35
**Laugh**	89.29 (36.1)	86.27 (34.1)	3.02 (-3.90;9.93)	0.39	3.73 (-3.25;10.71)	0.29
Girls	92.24 (34.3)	88.33 (35.0)	3.91 (-5.86;13.68)	0.43	3.89 (-6.03;13.82)	0.44
Boys	86.59 (37.6)	84.14 (33.1)	2.45 (-7.41;12.30)	0.63	3.60 (-6.58;13.78)	0.49
**Sit with support**	105.25 (42.6)	105.66 (40.4)	-0.41 (-8.76;7.94)	0.92	-0.58 (-9.02;7.85)	0.89
Girls	109.21 (43.7)	107.70 (40.5)	1.51 (-10.31;13.33)	0.80	2.12 (-9.86;14.10)	0.73
Boys	101.30 (41.3)	103.36 (40.4)	-2.06 (-13.92;9.80)	0.73	-3.14 (-15.52;9.24)	0.62
**Chest up**	106.41 (45.7)	102.31 (46.6)	4.10 (-5.54;13.73)	0.40	5.33 (-4.37;15.03)	0.28
Girls	108.85 (48.3)	105.15 (47.8)	3.67 (-10.49;17.88)	0.61	3.78 (-10.84;18.39)	0.61
Boys	104.12 (43.3)	99.16 (45.3)	4.96 (-8.22;18.14)	0.46	8.68 (-4.75;22.11)	0.20
**Roll over**	131.44 (47.8)	131.75 (46.3)	-0.32 (-9.14;8.51)	0.94	-0.39 (-9.28;8.50)	0.93
Girls	130.79 (53.2)	126.82 (44.2)	3.97 (-9.04;16.98)	0.55	4.62 (-8.52;17.76)	0.49
Boys	132.00 (42.8)	137.26 (48.2)	-5.26 (-17.30,6.78)	0.39	-6.07 (-18.50;6.36)	0.34
**Sit alone**	201.24 (35.6)	197.80 (30.7)	3.45 (-2.65;9.54)	0.27	3.40 (-2.73;9.53)	0.28
Girls	199.94 (37.0)	198.21 (26.7)	1.74 (-6.70;10.17)	0.69	2.21 (-6.38;10.79)	0.61
Boys	202.39 (34.3)	197.36 (34.4)	5.03 (-3.85;13.91)	0.27	4.75 (-4.29;13.79)	0.30
**Pincer grasp**	251.37 (50.5)	244.95 (50.4)	6.43 (-4.31;17.16)	0.24	5.89 (-4.88;16.67)	0.28
Girls	245.17 (49.8)	237.84 (45.1)	7.33 (-7.04;21.70)	0.32	8.63 (-6.07;23.33)	0.25
Boys	257.36 (50.7)	252.22 (54.5)	5.14 (-10.75;21.03)	0.52	1.82 (-14.79;18.42)	0.83
**Stand with help**	273.56 (59.0)	271.77 (62.7)	1.80 (-9.73;13.32)	0.76	1.20 (-10.32;12.73)	0.84
Girls	282.34 (64.0)	272.38 (61.8)	9.96 (-7.37;27.29)	0.26	9.74 (-7.68;27.15)	0.27
Boys	266.03 (53.5)	271.18 (63.8)	-5.16 (-20.56;10.25)	0.51	-5.86 (-21.55;9.83)	0.46
**Crawl**	280.53 (60.0)	284.83 (57.1)	-4.30 (-14.94;6.34)	0.43	-4.29 (-15.04;6.47)	0.43
Girls	285.59 (66.2)	281.88 (58.4)	3.71 (-12.47;19.89)	0.65	3.46 (-12.93;19.85)	0.68
Boys	276.05 (53.9)	287.94 (55.8)	-11.89 (-25.90;2.12)	0.10	-11.68 (-25.98;2.62)	0.11
**Walk with help**	318.98 (55.3)	317.36 (66.8)	1.62 (-9.53;12.78)	0.78	2.20 (-9.01;13.42)	0.70
Girls	324.25 (56.4)	320.83 (64.9)	3.42 (-12.44;19.28)	0.67	2.32 (-13.81;18.45)	0.78
Boys	314.27 (54.1)	313.70 (68.8)	0.57 (-15.21,16.34)	0.94	1.82 (-14.13,17.77)	0.82
**Stand alone**	349.71 (63.3)	355.83 (66.4)	-6.12 (-18.34;6.10)	0.33	-5.92 (-18.23;6.40)	0.35
Girls	358.7 (67.7)	355.29 (66.0)	3.39 (-14.72;21.49)	0.71	2.63 (-15.65;20.91)	0.78
Boys	341.97 (58.4)	356.40 (67.1)	-14.43 (-30.99;2.13)	0.09	-14.57 (-31.67;2.53)	0.10
**Walk alone**	398.61 (57.1)	399.84 (63.6)	-1.24 (-11.76;9.28)	0.82	-0.87 (-11.43;9.70)	0.87
Girls	404.31 (57.0)	401.62 (63.9)	2.69 (-12.43;17.81)	0.73	1.96 (-13.27;17.18)	0.80

© 2020 Sass L et al. *JAMA Network Open.*

Boys	393.58 (57.0)	397.95 (63.5)	-4.37 (-19.12;10.37)	0.56	-4.96 (-19.77;9.84)	0.51

Abbreviations: N=number, SD=standard deviation, CI=confidence interval, PC1=principal component 1, PC2=principal component 2.

[a] Data are expressed as mean (SD) with effect being differences in means (95% CI).

[b]Adjusted for maternal pre-intervention serum vitamin D_3 levels, n-3 long-chain polyunsaturated fatty acid RCT allocation, season of birth, and for overall analyses additionally for sex. Three mothers are missing pre-intervention whole blood vitamin D results.

© 2020 Sass L et al. *JAMA Network Open.*

eTable 3. Overview of Children at Each Neurodevelopmental Assessment

	N (%)	time	Assessment method	Description of differences in the number of children
**All children in study**	551 (100)	Birth		
**13 motor milestones**	520 (94.4)	0-2 years	Denver Developmental Index & Who registration form	- Not all parents filled out the registration form
**language test (word production)**	284 (51.1)	1 year	MacArthur-Bates Communicative Developmental Inventories	- Exclusion of children, with an additional language (other than Danish) spoken at home<br>- Late implementation of test
**language test (word production)**	393 (71.3)	2 years	MacArthur-Bates Communicative Developmental Inventories	- Exclusion of children, with an additional language (other than Danish) spoken at home
**Cognitive score**	503 (91.3)	2.5 years	Bayley Scales of Infant and Toddler Development, Bayley III	- Not all children participated in the 2.5-year visit
**General neurodevelopment**	405 (73.5)	3 years	Ages and Stages Questionnaire	- Late implementation of test
**Behavioral & emotional problems**	496 (90)	6 years	Strengths and Difficulties Questionnaire	- Not all children participated in the 6-year visit

© 2020 Sass L et al. *JAMA Network Open.*

eTable 4. Descriptive Classification of the Cognitive Composite Score of the Bayley-III

Cognitive composite score	Classification
≥ 130	Very superior
120-129	Superior
110-119	High average
90-109	Average
80-89	Low average
70-79	Borderline
≤ 69	Extremely low

© 2020 Sass L et al. *JAMA Network Open.*

eTable 5. Effect of High-Dose Vitamin D_3 Supplementation During Pregnancy on Scores From the Ages and Stages Questionnaire-3[a]

	**High-dose Vitamin D_3**	**Standard dose Vitamin D_3**	**Unadjusted**
	Median (IQR)[a]		P value
	N=197	N=208	
**Communication**	50 (50-55)	50 (50-55)	0.62
Girls	50 (50-55)	50 (50-55)	0.82
Boys	50 (50-55)	50 (50-55)	0.73
**Gross motor skills**	60 (55-60)	60 (55-60)	0.19
Girls	57.5 (55-60)	60 (55-60)	**0.04**
Boys	60 (55-60)	60 (55-60)	0.94
**Fine motor skills**	55 (50-60)	54.5 (45-60)	0.47
Girls	55 (50-60)	55 (50-60)	0.61
Boys	50 (45-60)	50 (40-60)	0.36
**Problem solving**	55 (50-60)	55 (50-60)	0.37
Girls	55 (50-60)	55 (50-60)	0.45
Boys	55 (46.25-60)	55 (50-60)	0.64
**Social skills**	55 (50-60)	55 (50-60)	0.68
Girls	60 (55-60)	60 (55-60)	0.95
Boys	55 (50-60)	55 (50-60)	0.79

Abbreviations: IQR = inter quartile range,
[a] Effect is expressed as median, with (IQR)

© 2020 Sass L et al. *JAMA Network Open.*

eTable 6. Effect of the High-Dose Vitamin D_3 Supplementation During Pregnancy on the Cognitive Composite Score of the Bayley-III Test Including Children Born Before 37 Gestational Weeks, Children With Low Birth Weight, and Children With a Neurological Diagnosis[a]

**Outcome**	**High-dose Vitamin D_3**	**Standard dose Vitamin D_3**	**Unadjusted β-coefficients**		**Adjusted[b] β-coefficients**	
	N=258	N=269	N=527		N=525	
	Mean (SD)	Mean (SD)	Effect (95% CI)	P value	Effect (95% CI)	P value
**Composite score**	104.6 (9.4)	104.4 (9.4)	0.15 (-1.45, 1.75)	0.86	0.18 (-1.43, 1.79)	0.83
**Girls**	105.4 (10.2)	105.4 (9.6)	-0.03 (-2.45, 2.40)	0.98	0.07 (-2.41, 2.54)	0.96
**Boys**	103.8 (9.3)	103.4 (8.3)	0.46 (-1.65, 2.57)	0.67	0.77 (-1.34, 2.89)	0.47

Abbreviations: N=number, SD=standard deviation, CI=confidence interval

[a] Data are expressed as mean (SD) with effect being differences in means (95% CI)..

[b] Adjusted for maternal pre-intervention serum vitamin D_3 levels, n-3 long-chain polyunsaturated fatty acid RCT allocation, season of birth, and for overall analyses additionally for sex. Two mothers are missing pre-intervention serum vitamin D_3 levels

© 2020 Sass L et al. *JAMA Network Open.*

eTable 7. Language Development in Women Not Participating in the n-3 LCPUFA Trial

	High-dose vitamin D_3	Standard dose vitamin D_3	Unadjusted	Adjusted[a]
	Median (IQR)		P value	P value
**1-year language test**				
	N=76	N=71	N=147	N=147
**Word production 1 year**	1.5 (0-5.25)	3 (1-6.5)	0.07	0.18
**2-year language test**				
	N=103	N=98	N=201	N=201
**Word production 2 years**	227.0 (79.0-347.5)	239.0 (116.2-411.2)	0.07	0.17

© 2020 Sass L et al. *JAMA Network Open.*

eFigure. Biplot From Principal Component Analysis of All 13 Milestones
Principal component 1 and 2 (PC1 and PC2) explain 37 and 16 % of the overall variation in the data, respectively

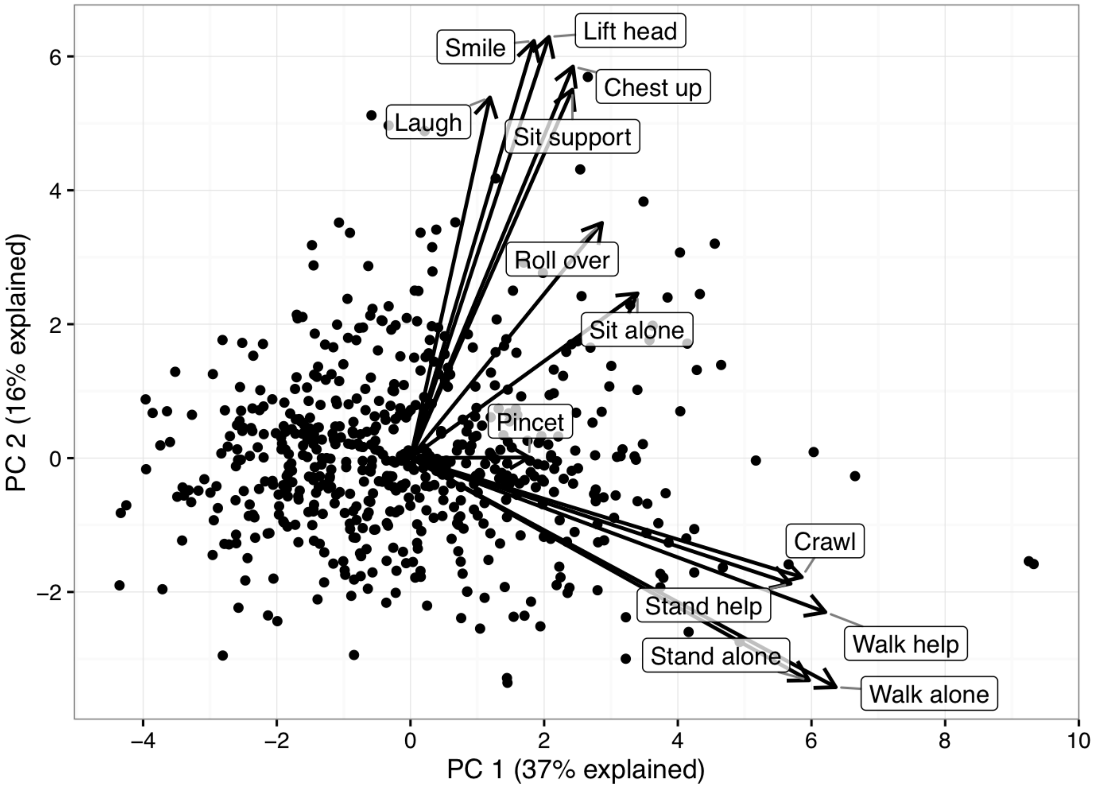

© 2020 Sass L et al. *JAMA Network Open.*
