# Supplementary material for: High-Dose Vitamin D Supplementation in Pregnancy and Neurodevelopment in Childhood: A Prespecified Secondary Analysis of a Randomized Clinical Trial
Source: JAMA Netw Open. 2020 Dec 8;3(12):e2026018. doi: 10.1001/jamanetworkopen.2020.26018 (PMC7724557; doi:10.1001/jamanetworkopen.2020.26018)
Supplement: Supplement 3. — Data Sharing Statement [file jamanetwopen-e2026018-s003.pdf]

Data Sharing Statement

Sass. High-dose Vitamin D Supplementation in Pregnancy and Neurodevelopment in Childhood. *JAMA Netw Open*. Published December 08, 2020. doi:10.1001/jamanetworkopen.2020.26018

Data
Data available: Yes
Data types: Deidentified participant data
How to access data: Deidentified individual participant data will be available in anonymous form upon request to the corresponding author Hans Bisgaard at bisgaard@copsac.com.
When available: With publication

Supporting Documents
Document types: None

Additional Information
Who can access the data: De-identified individual participant data will be available in anonymous form upon request to the corresponding author Hans Bisgaard at bisgaard@copsac.com.
Types of analyses: De-identified individual participant data will be available in anonymous form upon request to the corresponding author Hans Bisgaard at bisgaard@copsac.com.
Mechanisms of data availability: After approval of proposal.
